# Incorporation of Protein Hydrolysate into Rapeseed Meal-Based Materials to Improve Flexibility

**DOI:** 10.3390/polym17131740

**Published:** 2025-06-22

**Authors:** Sara Aquilia, Claudia Bello, Michele Pinna, Sabrina Bianchi, Walter Giurlani, Francesco Ciardelli, Luca Rosi, Anna Maria Papini

**Affiliations:** 1 Interdepartmental Research Unit of Peptide and Protein Chemistry and Biology, University of Florence, Via della Lastruccia 13, I-50019 Sesto Fiorentino, Italy; sara.aquilia@unifi.it (S.A.); claudia.bello@unifi.it (C.B.); 2 Department of Chemistry “Ugo Schiff”, University of Florence, Via della Lastruccia 13, I-50019 Sesto Fiorentino, Italy; walter.giurlani@unifi.it; 3 Spin-PET S.r.l., Viale R. Piaggio 32, I-56025 Pontedera, Italy; pinna@spinpet.it (M.P.); bianchi@spinpet.it (S.B.); ciardelli@spinpet.it (F.C.)

**Keywords:** biomaterials, rapeseed meal, rapeseed meal-based material, protein-based material, protein hydrolysate, plasticizer, flexibility

## Abstract

This study investigates the potential of rapeseed meal (RM), a protein-rich by-product of the rapeseed oil industry, as a raw material for the development of renewable materials. Due to the presence of antinutritional compounds, rapeseed meal is underutilized, primarily in low-value applications such as animal feed. In this work, rapeseed meal protein hydrolysates were enzymatically obtained and incorporated as plasticizers into rapeseed meal-based materials to improve their mechanical properties, water permeability, and thermal stability. Collagen hydrolysate has also been utilized as a low-cost additive to further enhance the material performance. The glycerol content was reduced to address permeability and migration issues associated with hydrophilic plasticizers. The results demonstrated that the incorporation of hydrolysates into rapeseed meal-based materials modulated thermal stability, water permeability, and mechanical properties—particularly elongation at break and flexibility. The latter increased proportionally with the hydrolysate content of RM-based materials. Additionally, aerobic biodegradation behavior, thermogravimetric analysis (TGA), and differential scanning calorimetry (DSC) supported the material’s favorable performance characteristics, highlighting the potential of rapeseed meal as a viable, biodegradable alternative for sustainable materials in industrial applications.

## 1. Introduction

Plastic materials have provided significant benefits to society due to wide-ranging applications and low costs. However, uncontrolled production and inadequate waste management have led to severe environmental problems, including the accumulation of plastic waste and microplastics in landfills and marine environments [1,2]. Therefore, developing sustainable alternatives to traditional fossil-based plastic has become a pressing challenge. In recent years, bioplastics derived from renewable and biodegradable resources have emerged as promising solutions for a more sustainable and circular management of natural resources [3,4,5,6].

Among these, protein-rich by-products of the food and agriculture industries, such as gluten meal, dairy whey, chicken feathers, and soy curd, are available in large quantities and have been successfully transformed into sustainable bioplastics [7,8].

Rapeseed meal is a promising by-product obtained from rapeseed oil extraction. The use of this meal is currently limited to low-value animal feed, and it is more frequently treated as waste and subjected to incineration or landfill disposal [6]. Additionally, rapeseed meal is unsuitable for use as a food ingredient due to the presence of some antinutritional compounds, including glucosinolates, erucic acid, phytates, and phenolic compounds, which negatively affect its palatability and digestibility [9]. Recently, the potential use of rapeseed meal as a raw material to produce cosmetics and bioplastics has gained attention due to its high protein content and cost-effectiveness [10]. Thermochemical methods, such as microwave-assisted pyrolysis, also offer real promise for the recovery of certain valuable chemicals, such as furfuryl alcohol or aromatic compounds, as well as for energy utilization [11].

Thus, rapeseed proteins represent a promising, environmentally sustainable alternative for the production of various technical products, including polymers, coatings, adhesives, detergents, and lubricants [12]. Despite its potential, the development of commercially suitable industrial products from raw rapeseed meal remains quite limited [13,14,15], with most studies focusing on bioplastics derived from protein isolates and on wet casting techniques, which are affected by scalability issues [16,17,18,19].

One of the main challenges in developing protein-based materials, including those derived from rapeseed, is their poor mechanical properties, slow biodegradability, and high water permeability due to the inherent structural and chemical nature of the proteins [7,20,21,22]. To address these issues, additives, such as plasticizers, are usually integrated into biomaterials. Plasticizers are low-molecular-weight, non-volatile substances that reduce inter- and intra-molecular interactions along the protein polymer chains enhancing chain mobility, increasing spacing between the polymer molecules, and reducing the proportion of crystalline regions relative to amorphous ones [23,24,25]. Common plasticizers used in protein-based materials include glycerol, sorbitol, polyethylene glycol, and water due to their compatibility with the biopolymer chains and their ability to form a new dispersed domain increasing material heterogeneity. Despite these advantages, such plasticizers are highly hydrophilic and hygroscopic, properties that can lead to migration issues and increased water permeability of the resulting material [23,26,27,28]. Hydrophobic plasticizers, such as fatty acids, waxes, vegetable oils, and citrate esters, can also be utilized. However, their low compatibility with proteins often results in phase separation, negatively impacting the material mechanical properties [29]. Active research is ongoing to develop alternative biocompatible plasticization strategies for protein-based materials [30].

Amino acids, being inherently compatible with proteins, can enhance structural mobility due to their low molecular weight, thereby increasing plasticity [31]. Notably, proline has been shown to be an effective and less toxic plasticizer for starch-based materials, significantly reducing the glass transition temperature of biomaterials [32,33].

Protein hydrolysates, consisting of low-molecular-weight peptides, have also emerged as promising plasticizers due to their strong compatibility with polymer matrices [34]. Several studies have demonstrated the plasticizing effects of hydrolysates from sources such as gelatin [28], wheat gluten [34,35], whey protein [35], and zein protein [27] on protein-based materials. These hydrolysates have been shown to lower the Young’s modulus and decrease the glass transition temperature, significantly improving the material mechanical properties and its processability [34].

The objective of the present study is to explore the use of rapeseed meal as a matrix for the development of cost-effective, bio-derived composite materials with potential industrial applications. We previously reported a novel method for the preparation of a partially flexible bio-composite derived from raw, protein-rich rapeseed meal, stabilized using appropriate interactive additives via compression molding [19]. To enhance the mechanical properties and thermal stability of the composite, we now incorporated rapeseed meal protein and collagen hydrolysate as key components, investigating their role as plasticizers. Moreover, the incorporation of protein hydrolysates can reduce the reliance on hydrophilic plasticizers like glycerol, thereby improving water permeability and minimizing migration issues while also maintaining the required flexibility. We successfully set up the enzymatic process with Alcalase^®^ on the raw meal without any other previous purification and scaled up the enzymatic hydrolysis of rapeseed meal protein, proving that the process can be amenable to industrial application. The incorporation of crosslinking agents and protein hydrolysates allows for the modulation of the mechanical, thermal, and water-resistant properties of the composites toward specific applications. These results highlight that composite materials based on rapeseed meal can exhibit physical and mechanical properties that may be suitable for potential industrial applications in replacement for fossil-based materials.

## 2. Materials and Methods

### 2.1. Materials

The rapeseed meal (RM) was generously supplied by Italcol S.p.A. (Castelfiorentino, FI, Italy). Alcalase^®^ 2.4 L was kindly provided by Novazymes (Bagsværd, Denmark). Proline and collagen hydrolysate were purchased from Nutrivita shop (Lake Forest, CA, USA). Rennet casein was provided by Fontana Enzo s.r.l. (Sarmato, PC, Italy) Glycerol, poly-(ethylene glycol) diglycidyl ether, sodium sulfite (Na_2_SO_3_), sodium dodecyl sulfate (SDS), guanidinium chloride, 2-mercaptoethanol, Tris-HCl, acrylamide, Coomassie Blue, methanol, acetic acid, acetonitrile (ACN), and trifluoroacetic acid (TFA) were purchased from Merck (Darmstadt, Germany). All the reagents were of analytical or reagent grade and were employed without additional purification.

### 2.2. Enzymatic Proteolysis of Rapeseed Meal and Scale-Up

Rapeseed meal (5% *w*/*v*, protein basis, Table S1 Supporting Information) was suspended in deionized water and the pH was adjusted to 8 with 1 M NaOH. To denature proteins and enhance their accessibility to the enzyme, the slurry was then heated to 50 °C and stirred at 140 rpm for 30 min using a C24 incubator shaker (Edison, NJ, USA) prior to the addition of the proteolytic enzyme. Alcalase^®^ 2.4 L was added to the slurry at an enzyme/substrate ratio (E/S) of 1:25 protein basis. Digestion was carried out at the above-mentioned conditions for 6 h, maintaining the pH constant with 1M NaOH. After digestion, the enzyme was inactivated by adjusting the reaction mixture to pH 4.0 with 1M HCl followed by immersing the reaction vessel in a boiling water bath for 10 min. The slurry was centrifuged at 4000 rpm for 35 min and the supernatant was collected and freeze-dried.

The protein concentration in the hydrolysate was determined using the Lowry method [36] and the degree of hydrolysis (DH) was calculated on the basis of the following equation (Equation (1) [34]:

(1) $$\mathrm{DH}=\left(\;\frac{\mathrm{hs}-h0}{\mathrm{ht}-h0}\right)\;\times \;100$$

where hs and h0 are the terminal -NH_2_ concentrations of hydrolyzed and non-hydrolyzed protein, respectively, and ht is the number of amide bonds in the protein substrate (7.8 equiv/g for rapeseed protein) [37].

Following this, a ten-fold scale-up was conducted under identical conditions of pH and temperature, maintaining the same E/S and incubating for 1.5 h.

### 2.3. Polyacrylamide Gel Electrophoresis

The molecular mass distribution of the initial protein fraction and hydrolysate was analyzed by sodium dodecyl sulfate–polyacrylamide gel electrophoresis (SDS-PAGE) using a Bio-Rad Mini-PROTEAN^®^ 3 cell system (Bio-Rad, Hercules, CA, USA). A 4% acrylamide stacking gel (pH 6.8) and a 16% separating gel (pH 8.8) were employed. Samples were prepared at a concentration of 1 mg/mL in deionized water, centrifuged (4000 rpm, 10 min) to remove any insoluble material, and subsequently mixed at a 1:1 ratio with Laemmli sample buffer (4% SDS, 20% glycerol, 10% 2-mercaptoethanol, 0.004% bromophenol blue, 0.125 M Tris, and pH 6.8). The mixtures were heated in a water bath at 96 °C for 10 min. An aliquot of 15 μL from each sample was loaded onto the gel. The peptide bands were stained in a solution of 5% Coomassie Blue in methanol/acetic acid/water (4:0.8:5.2) for 3 h, followed by destaining in a methanol/acetic acid/water solution (4:0.8:5.2) for 12 h. Thermo Scientific PageRuler Prestained Protein Ladder (10–180 KDa) (Thermo Fisher Scientific, Waltham, MA, USA) and Spectra™ Multicolor Low Range Protein Ladder (1.7–40 KDa) (Thermo Fisher Scientific, Waltham, MA, USA) were used as a reference for calibrating the molecular weight of the peptides.

### 2.4. Chromatographic Analysis of Rapeseed Meal Hydrolysates

The hydrophilic properties of the peptides derived from rapeseed meal hydrolysate (RMH) were investigated by reverse-phase high-performance liquid chromatography (RP-HPLC). The freeze-dried hydrolysate was solubilized in 5% ACN and the peptide mixtures were analyzed employing an Alliance Waters 2695 HPLC coupled to a Photodiode Array Detector Waters 2996 (Waters, Milford, MA, USA), equipped with a Supelco BIOshell A160 Peptide C18 column (10 cm × 3.0 mm, 2.7 μm) (Supelco, St. Louis, MO, USA), using solvent systems A (0.1% TFA in H_2_O) and B (0.1% TFA in ACN). Elution was performed using a 15 min linear gradient from 5% to 95% B at a flow rate of 0.6 mL/min and a temperature of 35 °C. The eluates were monitored at wavelengths of 214 nm and 280 nm.

### 2.5. Rapeseed Meal-Based Material Preparation

The rapeseed meal was dry milled at 1800 rpm for 15 min at room temperature. The resulting powder was placed in an oven overnight at 50 °C to remove moisture. Next, 40 g of rapeseed meal (53% w/wt) was mixed with 10 g of a protein-binding agent (Rennet casein, 13 w/wt%), 20 g of glycerol (27% w/wt), 10 g of 20% Na_2_SO_3_ in 1M guanidine hydrochloride solution (14% w/wt), and 2.5 g of poly-(ethylene glycol) diglycidyl ether (2 × mol lysine-based, 2.86% of the full amino acid content) using a blender blade for 15 min to obtain a homogeneous blend. Subsequently, the mixture was heated to 150 °C for 15 min and pressed using a flat-bed press (CAMPANA model: PRESSA PM20/200, Milano, Italy) at 150 °C for 20 min at 250 Bar (mold dimensions: 8 cm × 16 cm × 1.5 mm). The resulting material (RM-mat, Table 1) was then cooled to room temperature and stored in a desiccator.

Modified rapeseed meal specimens (see Table 1 for detailed composition) were prepared according to the previously described protocol, with the inclusion of collagen hydrolysate (CH) and rapeseed meal hydrolysate (RMH) at concentrations of 0.25%, 0.50%, 0.75%, and 1.00% (*w*/*w*). Modified specimens are labeled as C1 to C4 for collagen hydrolysate and A1 to A4 for rapeseed meal hydrolysate.

Additionally, starting from materials C3 and A3, the glycerol content was decreased from 27% to 10% in specimen G1 to G3 and G4 to G6, respectively (Table 2). Materials G1P4 and G4P4 were formulated using 20% glycerol (*w*/*w*), 7% proline (*w*/*w*), and 0.75% collagen and rapeseed meal hydrolysate, respectively (see Table 2 for detailed composition).

### 2.6. Thickness

The thickness of the specimen was measured using a handheld micro-meter (B.C. Ames Co., Waltham, MA, USA). Three measurements were taken at the following points on the RM-based materials: the center and the right and left sides, 2 cm from the edge. The average of these measurements was calculated and used to compute tensile strength and elongation.

### 2.7. Mechanical Properties

After pressing, the rapeseed meal specimen was kept at 25 °C and 75% relative humidity for 24 h and then cut into three or four standard dumbbell-shaped molds, each measuring 60 mm (length) × 5 mm (width) × 3 mm (thickness). The mechanical properties were evaluated using a Shimadzu instrument (Shimadzu, Model AGS-X 5 KN, Milano, Italy) following the Standard Test Method for Tensile Properties of Plastics (ASTM D638-91) [38]. The testing speed was set at 5 mm/min. Tensile strength, elongation at break (%), and elastic modulus were determined based on measurements taken from at least three replicates.

### 2.8. Water Absorption Measurements

The water absorption was determined following the Standard Test Method for Water Absorption of Plastics (ASTM D570-81) [39]. The RM samples were pre-conditioned at 50 °C for 24 h and subsequently cooled to room temperature before being weighed (W1). Then, they were immersed in distilled water at 25 °C for 24 h. After this time, they were removed from the water, dried with a paper towel, and then weighed (W2). The water absorption was calculated as a percentage of the initial weight (Equation (2)) using the following equation:

(2) $$\mathrm{Increase}\;\mathrm{in}\;\mathrm{weight}\;\left(\%\right)=\left(\frac{W2-W1}{W1}\right)\;\;100$$

To assess the soluble material loss, as shown in the following equation, the specimen was weighed after being dried in an oven at 50 °C for an additional 24 h (W3) (Equation (3)):

(3) $$\mathrm{Soluble}\;\mathrm{matter}\;\mathrm{loss}\;\left(\%\right)=\left(\frac{W1-W3}{W1}\right)\;\;100$$

The total water absorption for 24 h was calculated considering the soluble material loss, using the following formula (Equation (4)):

(4) $$\mathrm{Total}\;\mathrm{water}\;\mathrm{absorption}\;\left(\%\right)=\left\{\frac{\left(W2+W3\right)-W1}{W1}\right\}\;\;100$$

where W1 is the initial sample weight, W2 the sample weight after 24 h, and W3 is the weight of soluble material. The tests were conducted in triplicate.

### 2.9. Hydrolysate Migration Test

The release of collagen and rapeseed meal protein hydrolysate from plasticized material was evaluated by reverse-phase ultra-high-performance liquid chromatography (UHPLC). Specimens were immersed in distilled water at 25 °C for 24 h and in 10% ethanol at 40 °C for 10 days. After this period, the solutions were analyzed by an UHPLC Thermo Dionex UltiMate 3000 (Thermo Fisher Scientific, Walthman, MA, USA) equipped with an Acquity UPLC BEH C18 column (1.7 μm, 2.1 × 50 mm, Des Moines, IA, USA) using solvent systems A (0.1% formic acid in H_2_O) and B (0.1% formic acid in ACN). Elution was performed with an 8 min linear gradient from 1% to 95% phase B at a flow rate of 0.5 mL/min at 35 °C. The eluates were monitored at 214 nm to detect the presence of proteins or peptides in the eluate.

### 2.10. Thermogravimetric Analysis

The thermal stability of the protein-based materials was assessed via thermogravimetric analysis (TGA) conducted utilizing a Perkin Elmer 4000 instrument (Waltham, MA, USA). The samples were analyzed using a nitrogen flow rate of 30 mL/min with heating from 25 to 815 °C at 10 °C/min. DTG curves were smoothed using a second-order polynomial over six points to preserve minor but relevant thermal features that could be lost with stronger smoothing.

### 2.11. Differential Scanning Calorimetry

The glass transition temperature and melting temperature of the RM-based specimens were measured using a Waters Discovery DSC 2500 (TA Instrument, New Castle, DE, USA) following the Standard Test Method for Transition Temperatures and Enthalpies of Fusion and Crystallization of Polymers by Differential Scanning Calorimetry (ASTM D3418−21) [40]. In the first cycle, about 10 mg of the samples were equilibrated at 0 °C and then heated to 160 °C at a rate of 10 °C/min, equilibrated at 160 °C for 1 min, and then cooled back to 0 °C. In the second cycle, the samples were heated to 250 °C at a rate of 10 °C/min. This procedure has been used previously for other biomaterials [8]. The glass transition temperature was found from the inflection in the heat flow versus temperature curve on the second heat cycle and the melting enthalpy was evaluated by integration of the areas under the melting peak using TRIOS v5.5.1.5 software (TA Instrument, New Castle, DE, USA).

### 2.12. Scanning Electron Microscopy

Scanning electron microscopy (SEM) analyses were performed with a variable pressure Hitachi SU3800 instrument (Hitachi High-Tech, Tokyo, Japan) equipped with an Ultim Max 40 silicon drift EDS detector and AZtecLive 6.1 software (Oxford Instruments NanoAnalysis, Abingdon, UK). The measurements were performed at progressively increasing magnifications with an accelerating voltage of 15 kV. Before the SEM analyses, the samples were metalized using a SC7640 Polaron Sputter Coater with an SC510-314B gold/palladium target (Quorum Technologies, Laughton, UK). The surface and cryofractured sections of the samples were analyzed. To ensure uniform cooling, the samples were immersed in liquid nitrogen for approximately 1 min. They were then split into two fragments using two forceps.

### 2.13. Aerobic Biodegradation Measurement

The biodegradation rates of G1, G4, G1P4, and G4P4 materials were measured according to the Standard Test Method for Determining Aerobic Biodegradation of Plastic Materials in Soil (ASTM D5988) [40]. Each sample was placed into a jar and mixed with soil at a 1:6 sample-to-dry compost ratio. A beaker containing 20 mL of 0.5N KOH and a beaker containing 50 mL of distilled water were both placed into each jar, and the moisture content in each jar was maintained by adding deionized water to counterbalance losses. The scheme of the biodegradation set up applied in this work is shown in Figure S7A,B (Supporting Information, page 8). The amount of CO_2_ emitted due to the decomposition of the material in each jar by microorganisms in the soil was determined by titration of residual KOH with 0.5 N HCl and phenolphthalein as an indicator using the following equation (Equation (5)):

(5) $$\mathrm{Released}\;{CO}_{2}\;(\mathrm{mg})=V\;\times \;N\times \;22$$

where V is the volume of consumed acid in the test specimen in the jar, N the concentration of the acid, and 22 is the number of equivalents of CO_2_. The titrations were performed for three consecutive months.

The theoretical amount of CO_2_ produced by the total oxidation of incubated samples in each flask can be calculated by the following equation (Equation (6)):

(6) $$\mathrm{Theoretical}\;{CO}_{2}\;(\mathrm{mg})\;=\frac{M\times \;C\times \;44}{12}$$

where C, M, 44, and 12 are the relative amount of total carbon per total weight of sample, the total weight of sample, the molar mass of CO_2_, and the molar mass of carbon.

Biodegradation was calculated as the percentage (%) of carbon in the biomaterial mineralized as CO_2_ according to the following equation (Equation (7)):

(7) $$\mathrm{Biodegradation}\;\left(\%\right)=\frac{\;\left({CO}_{2}\right)s-({CO}_{2})c}{\;({CO}_{2})t}\;\times 100$$

where (CO_2_)s and (CO_2_)c are the amount of CO_2_ produced in the sample and in the control, while (CO_2_)t is the theoretical amount of CO_2_.

## 3. Results and Discussion

### 3.1. Rapeseed Meal Protein Hydrolysate Characterization

Depending on factors such as cultivation conditions, harvesting techniques, and processing methods the protein content in rapeseed meal can vary, typically ranging from 35% to 40% [10].

**Figure 1:**
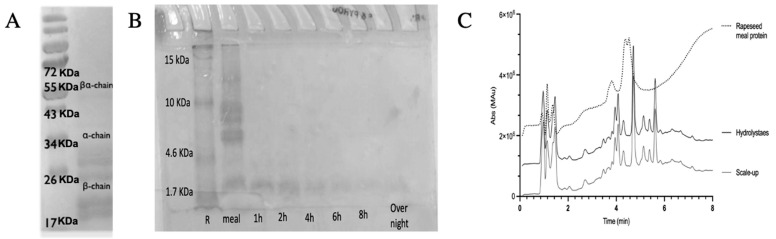
(**A**) Electrophoretic (SDS–PAGE) pattern of cruciferin (marker: Thermo Scientific PageRuler Prestained Protein Ladder (10–180 KDa) (Thermo Fisher Scientific, Waltham, MA, USA); (**B**) electrophoretic (SDS–PAGE) pattern of the rapeseed meal protein, rapeseed meal, and protein hydrolyzed by Alcalase^®^ over the time (marker: Spectra™ Multicolor Low Range Protein Ladder (1.7–40 KDa)) (Thermo Fisher Scientific, Waltham, MA, USA)); (**C**) RP-HPLC chromatogram of rapeseed meal protein, rapeseed meal protein hydrolysate (reaction time: 6 h), and scale-up of the enzymatic process. A 15 min linear gradient from 5% to 95% B (A (0.1% TFA in H_2_O) and B (0.1% TFA in ACN)) at 35 °C and flow rate of 0.6 mL/min.

The predominant proteins in rapeseed meals are cruciferin (12S globulin) and napin (2S albumin), representing approximately 60% and 20% of the total crude protein content, respectively [11]. Characteristic polypeptide bands of cruciferin were identified within the ca. 40–55 kDa and ca. 20–32 kDa ranges, as observed under reducing SDS-PAGE (Figure 1A). Napin, a protein with a molecular weight smaller than cruciferin, exhibited a characteristic polypeptide band of ca. 9–11 kDa under reducing conditions, consistent with previous studies [41] (Figure 1B).

Enzymatic digestion of rapeseed proteins in raw meals with Alcalase^®^ led to a hydrolysate that was characterized by SDS-PAGE and RP-HPLC. The final brownish color is probably due to the Maillard reaction, which might occur between some amino acid side chains and reducing sugars in the meal [42]. Alcalase^®^ is a commercial enzyme preparation commonly used for the hydrolysis of soy protein isolate on a semi-industrial scale. The main component is a serine protease of bacterial origin, first obtained from Bacillus subtilis [43]. Alcalase^®^ fully hydrolyzed rapeseed meal protein after 6 h mostly to a peptide of a molecular weight lower than 1.7 KDa (Figure 1B). Electrophoresis revealed that the largest peptides, corresponding to the cruciferin subunits, were cleaved at the very beginning of the reaction, while the napin polypeptides were gradually degraded over time (Figure 1B). The hydrolysis time was determined empirically to achieve maximum hydrolysis without inducing peptide aggregation (Table S2, Supporting Information) [8]. The RP-HPLC profiles of rapeseed meal proteins are shown in Figure 1C (dotted line). The profile of the native proteins showed three main peak groups (Figure 1C). By comparison with patterns of pure napin and cruciferin [44], the first peak corresponds to the 2S albumins (napin); the two others correspond to the cruciferin polypeptides. The cruciferin and napin polypeptides disappeared completely in the hydrolysate (Figure 1C), forming mixtures of small-chain hydrophilic peptides (Figure S1, Supporting Information). However, according to the SDS-PAGE analysis, some non-hydrolyzed napin can be present under the broad peaks (Figure 1C).

Scaling laboratory protocols to an industrial level presents several challenges, including reagent consumption, reaction time, energy requirements, and labor costs [45]. To address these issues, a ten-fold scale-up of the hydrolysis protocol was conducted under identical pH and temperature conditions, maintaining the same enzyme-to-substrate (E/S) ratio. The reaction time was set to 1.5 h, as increased process efficiency is observed at larger scales [43]. The RP-HPLC chromatogram of the hydrolyzed mixture after the scale-up is presented in Figure 1C.

### 3.2. Rapeseed Meal-Based Materials

**Figure 2:**
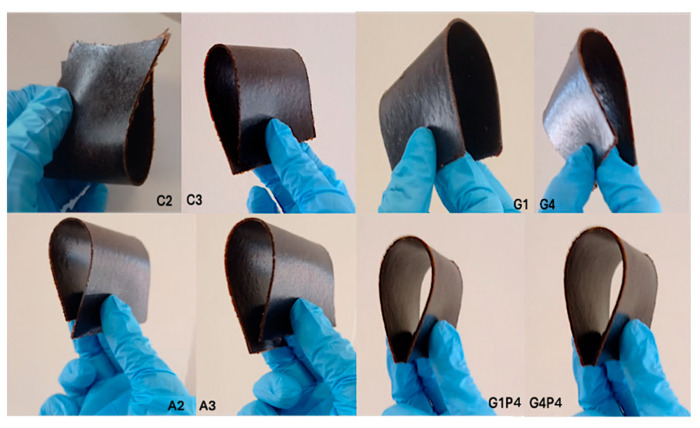
Visual appearance of rapeseed meal-based biomaterial with the incorporation of protein hydrolysate. From left to right: C2, C3, G1, G4, A2, A3, G1P4, and G4P4 (for specimen composition refer to Table 1 and Table 2).

Starting from our previous results [19], we prepared a structurally stable, partially flexible bio-composite via pressure molding (150 °C, 250 Bar) from raw rapeseed meal stabilized via crosslinks with selected epoxides. Controlled denaturation, induced by disulfide bond-reducing (Na_2_SO_3_) and denaturant agents, significantly increased entanglements and crosslinks between protein chains and the crosslinker (Epoxy-PEG) [19]. Then, the plasticizing effects of rapeseed proteins and collagen hydrolysates were systematically examined by incorporating these peptide mixtures at varying concentrations. These mixtures contain short-chain peptides with high molecular mobility, which, when integrated into the composite, reduce intermolecular forces and increase the free volume between polymer chains, thereby improving the material flexibility. Specifically, rapeseed meal protein hydrolysate was chosen because of its high compatibility with the material matrix. Moreover, Zhang et al. [46] showed that rapeseed protein hydrolysate improves mechanical properties and reduces the moisture permeability of chitose composites. On the other hand, collagen hydrolysate from the leather industry is a low-cost material acting as a plasticizer in blend with various synthetic polymers such as poly-(butylene succinate-co-adipate) (PBSA), polyvinyl alcohol (PVA), low-density polyethylene (LDPE), polyvinylchloride (PVC), and polycaprolactone (PCL) [47]. Furthermore, the use of protein hydrolysates as plasticizers may decrease the amount of the commonly used hydrophilic plasticizers needed in protein-based materials, thereby minimizing the permeability of the materials while producing needed flexibility [26]. Therefore, the glycerol content was reduced from 27% to 20% and replaced with proline to modulate the material permeability. All the materials produced were homogeneous, flexible, and exhibited the dark-brown color characteristic of rapeseed meal (Figure 2). Notably, this flexibility occurs without permanent deformation as the material fully recovers its original shape due to the elastic properties conferred by the crosslinking. The thickness of the films ranged from 0.2 to 0.3 mm, with substantial flexibility despite the stabilizing crosslinks provided by the epoxide.

### 3.3. Mechanical Properties

#### 3.3.1. Use of Collagen and Rapeseed Meal Hydrolysate

**Figure 3:**
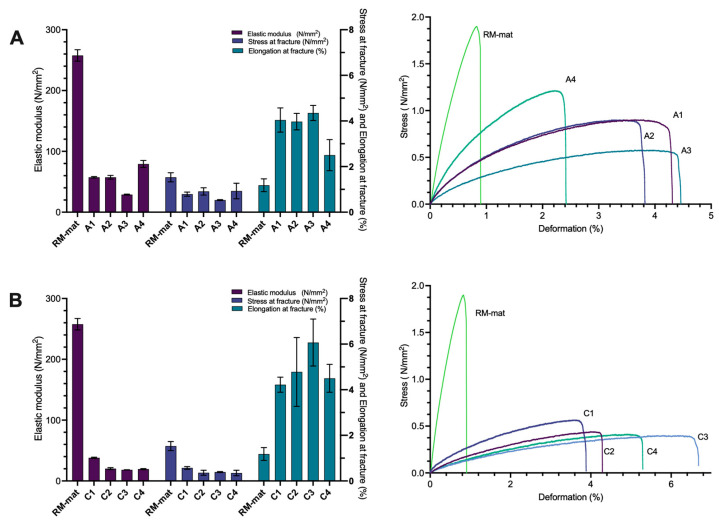
Histograms of tensile properties (**left**) and stress/strain diagrams (**right**) of RM-based materials with (**A**) the addition of collagen hydrolysate (material C1–C4); (**B**) rapeseed meal protein hydrolysate (material A1–A4). (For specimen composition, refer to Table 1) Data are presented as an average of three measurements ± of standard deviation. Error bars show standard deviation.

The mechanical properties of the materials modified by the addition of collagen (materials C1–C4) or rapeseed meal protein hydrolysate (materials A1–A4) at different concentrations were evaluated and compared to those of the control rapeseed meal-based material plasticized with glycerol (reference RM-mat). Specifically, the incorporation of collagen hydrolysate into the RM matrix resulted in a progressive decrease in both elastic modulus and tensile strength with increasing concentrations, particularly within the 0.25% to 0.75% range (Figure 3A and Table 3, materials C1–C3). Notably, the elongation at break for the C3 specimen was 6.07%, approximately six times greater than that of the control RM material (RM-mat), which may be attributed to the insertion of collagen hydrolysate chains between the protein macromolecules, enhancing flexibility and extensibility [28]. However, the material with 1% collagen hydrolysate (material C4) exhibited mechanical properties, including elastic modulus and tensile strength, comparable to material C3, while the elongation at break decreased to 4.50%. This reduction is likely due to the formation of additional hydrogen bonds, which restrict polymer chain mobility.

The rapeseed meal protein hydrolysate also demonstrated a notable effect on the rapeseed meal-based materials, as indicated by the reduction in Young’s modulus and tensile strength (Figure 3B and Table 4). Particularly, the A3 specimen, containing 0.75% hydrolysate, exhibited an elastic modulus approximately ten times lower and an elongation at break four times higher than that of the control RM material (79.31 N/mm^2^ and 4.35%, respectively). This suggests that the rapeseed meal hydrolysates weaken the hydrogen bonding network responsible for the rigidity of the RM material by integrating between polymer chains [8,27]. However, the mechanical properties of the materials A1-A4 exhibited low defined correlation between tensile strength and elastic modulus across different concentrations. This lack of a clear trend may be attributed to the inherent heterogeneity of the hydrolysate and the inherent multiphase composition of the RM, which is contrary to materials incorporated with collagen hydrolysate which tend to show more defined mechanical property relationships. Indeed, elongation occurs only if the composite material is sufficiently soft and the crosslinking is moderate, with crosslink bonds separated by at least 200 Å. In the absence of such energy-absorbing elongation, the composites may fracture at the interfaces between different domains.

The decrease in tensile strength in materials A1–A4 and C1–C4 compared to the reference RM-mat is in agreement with findings in the literature regarding the fabrication of protein-based bioplastics through heat treatment [5,8]. Notably, materials C1–C4—incorporating collagen hydrolysate, generally demonstrate superior plasticizing effects compared to those containing rapeseed meal protein hydrolysate (materials A1–A4). This is likely due to the more uniform peptide composition of collagen hydrolysate, which enhances its ability to disrupt interchain interactions and improves flexibility.

#### 3.3.2. Replacement of Glycerol with Proline and Use of Collagen and Rapeseed Meal Hydrolysate

**Figure 4:**
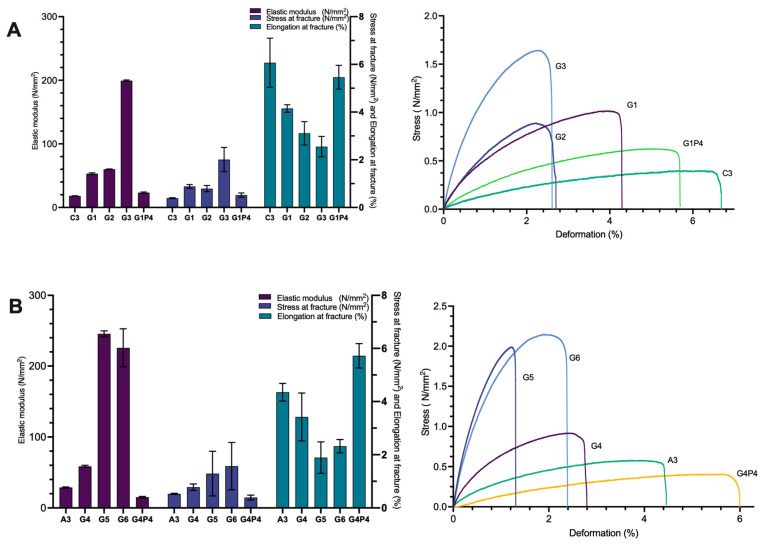
Histograms of tensile properties (**left**) and stress/strain diagrams (**right**) of rapeseed meal-based material with (**A**) decreased amount of glycerol and the addition of collagen hydrolysate (materials G1–G3) and proline (material G1P4); (**B**) rapeseed meal protein hydrolysate (material G4–G6) and proline (material G4P4) (for specimen composition refers to Table 2). Data are presented as an average of three measurements ± of standard deviation. Error bars show standard deviation.

The utilization of hydrolysate mixtures as plasticizers in protein-based materials offers the potential to reduce the required glycerol content, thereby decreasing permeability while maintaining the desired flexibility. Previous research has shown that hydrolyzed whey protein isolate (WPI) requires a lower amount of plasticizer compared to its unhydrolyzed form to produce films with optimal mechanical properties [23]. In light of this, we reduced the glycerol content in the C3 and A3 formulations (see Table 2). Notably, only the G1 and G4 formulations, which contained 20% glycerol, exhibited mechanical properties comparable to those of C3 and A3. When the glycerol concentration was reduced to 10% or 15% (material G2–G3 and G5–G6), the resulting mixtures became dry and difficult to process. This reduction in glycerol led to a ten-fold increase in the elastic modulus and a four-fold decrease in the elongation at break (Figure 4A,B, Table 5 and Table 6).

To mitigate these issues, 7% proline was added to the G1 and G4 formulations to compensate for the lower glycerol content. This modification resulted in a decrease in the elastic modulus to 23.23 N/mm^2^ for G1P4 and 15.08 N/mm^2^ for G4P4, while elongation at break increased to 5.46% and 6.72%, respectively.

### 3.4. Water Absorption Measurements

**Figure 5:**
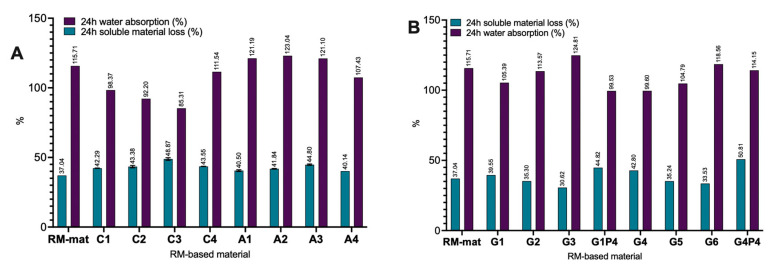
Water permeability of RM-based materials with (**A**) collagen (C1–C4) and rapeseed meal protein (A1–A4); (**B**) decreased amount of glycerol (G1–G3) and (G4–G5), and addition of proline (G1P4 and G4P4). Data are presented as an average of three measurements ± standard deviation.

Water permeability is a critical factor in evaluating the suitability of materials for industrial applications. The incorporation of collagen hydrolysate into RM-based materials resulted in significantly reduced water absorption compared to RM materials plasticized with glycerol. Specifically, increasing the concentration of collagen hydrolysate led to a progressive decrease in water absorption (Figure 5, materials C1–C3). This effect is attributed to the integration of collagen peptides into the protein matrix through hydrogen bonding, which enhanced the material structural density [46]. The C3 specimen, containing 0.75% collagen hydrolysate, exhibited the lowest water absorption value at 85.31%. However, when the collagen hydrolysate concentration was further increased to 1% (material C4), excess collagen hydrolysate molecules unbound within the protein network contributed to an increase in water permeability [46]. Conversely, rapeseed meal protein hydrolysate incorporation into RM material led to a significant increase in water absorption, reaching about 20% more than the material’s initial weight, independent of the hydrolysate concentration (Figure 5A, materials A1–A3). This is attributed to the hydrophilic nature of the peptides, which facilitated water molecule diffusion through the protein matrix, thereby increasing water permeability [48].

For materials G1–G3 and G4–G6, a decrease in glycerol content from 27% to 10% was associated with an increase in water permeability (Figure 5B). This suggests the insertion of hydrolysate peptides among protein chains, increasing the free volume within the matrix. As a result, water absorption was increased [26,28].

The soluble material loss remained constant at approximately 40% for materials A1–A4 and C1–C4 and decreased to around 30% in materials G3 and G6. Jiménez-Rosado et al. [49] reported that in addition to glycerol, a small amount of soluble protein can be lost during immersion due to the high solubility of rapeseed proteins. The possible migration of rapeseed meal protein or peptide hydrolysate from the material into the solution was evaluated by migration tests performed in water and 10% ethanol as a simulant food solution [35]. The RP-UPLC chromatograms (Figure S4) did not detect the presence of proteins or peptides, indicating that the hydrolysate forms strong hydrogen bonds within the protein matrix and does not lead to migration issues.

### 3.5. Thermogravimetric Analysis

**Figure 6:**
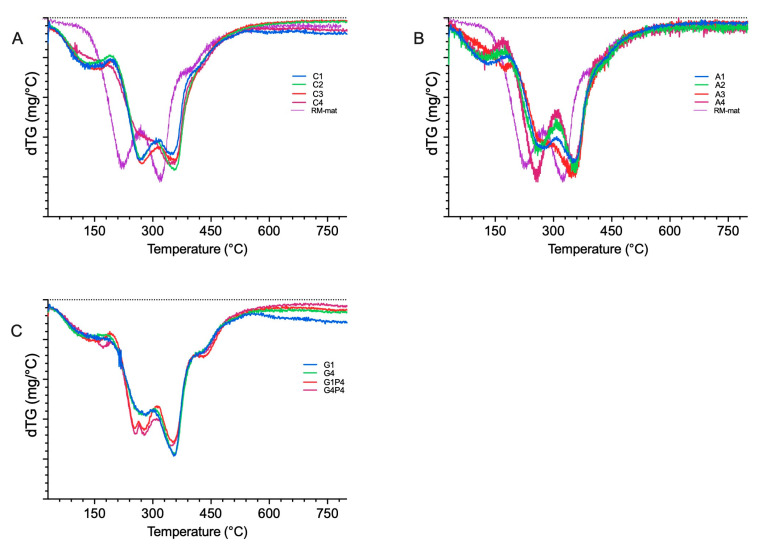
First-derivate curve of TGA of RM-based materials with: (**A**) the addition of collagen hydrolysate (material C1–C4), (**B**) rapeseed meal protein hydrolysate (materials A1–A4), (**C**) decreased amount of glycerol and the addition of collagen hydrolysate (material G1) and proline (material G1P4), and decreased amount of glycerol and rapeseed meal protein hydrolysate (material G4) and proline (material G4P4).

The thermal stability of the rapeseed meal-based materials was assessed using thermogravimetric analysis (TGA). The TGA and first-derivative curves (Figure S2 and Figure 6, respectively) show a characteristic weight loss profile for all samples, consistent with what was observed in other protein-based materials [50]. Specimen tested displayed higher thermostability compared to the reference material (RM-mat). The initial weight loss, occurring between 30 °C and ca. 150 °C, is attributed to the gradual moisture evaporation. This effect is particularly pronounced in the materials containing collagen hydrolysate (materials C1–C3, Figure 6B and Figure S3B) and rapeseed protein hydrolysate (materials A1–A4, Figure 6A and Figure S3A) due to their hydrophilic nature and ability to bind water molecules. The subsequent weight loss, observed between 150 °C and 250 °C, is due to the evaporation of plasticizers and the degradation of lower-molecular-weight peptides [47]. A delayed onset of thermal degradation in the plasticizer evaporation region was evident for materials C3 and A3, which is likely due to enhanced plasticizer–polymer interactions (Figure 6A,B), indicating a slight improvement in thermal stability [51]. Furthermore, materials G1P4 and G4P4 (Figure 6C and Figure S3C) exhibit an additional degradation step between 150 °C and 250 °C, corresponding to the thermal degradation of proline additive. Peptide bond cleavage occurs between 280 °C and 400 °C, with the degradation process extended over a prolonged period, which can be attributed to the presence of multiple protein fractions exhibiting a broad molecular weight distribution [17]. Additionally, material A4 shows an inversion of the area ratios between the second and third thermal degradation steps (Figure 6A), which may be attributed to an excess of unbound hydrolysate molecules.

### 3.6. Differential Scanning Calorimetry

Differential scanning calorimetry (DSC) was employed to investigate the effects of collagen and rapeseed meal protein hydrolysates and proline on the melting and crystallization behavior of RM-based materials. To avoid interference from prior thermal history, the first heating scan was omitted, and attention was focused on the second heating. The DSC curves for RM-based materials, C3, G1, G4, and G1P4, during the second heating are presented in Figure S6. From these curves, the glass transition temperature (Tg), melting temperature(s) (Tm), and melting enthalpy (ΔH) are obtained, and the results are summarized in Table 7.

The A3 and G4P4 specimen melted during the first heating cycle, preventing the evaluation of their thermal behavior. No significant changes in Tg were observed compared to the reference material RM-mat (Figure S5B Supporting information, page 6). Domains containing lower-molecular-weight species as plasticizers can provide additional phases with very low Tg not detected here.

On the other hand, the 20% increase in melting enthalpy in all materials probably results from the easier protein crystallization related to the lower internal viscosity produced by the hydrolysate (Figure S5A Supporting information, page 6).

### 3.7. Scanning Electron Microscopy

**Figure 7:**
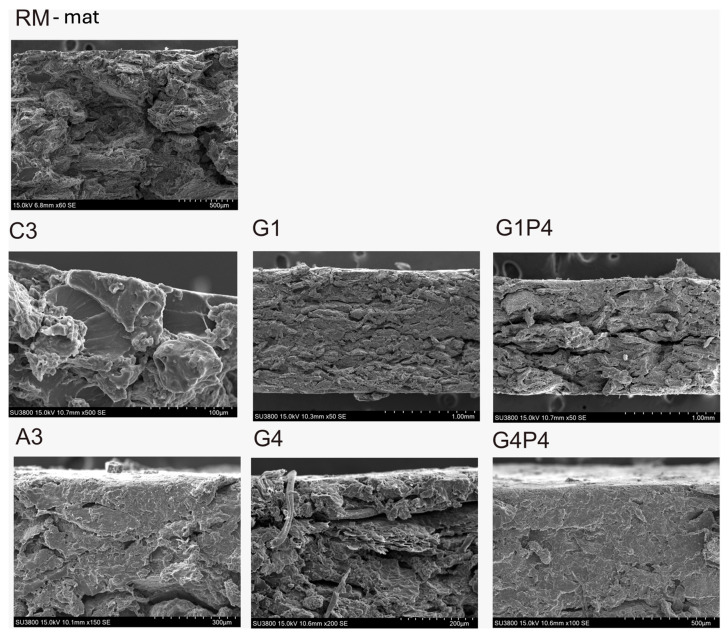
Scanning electron microscopy (SEM) images of cryofracture cross-section of specimen RM-mat, C3, A3, G1, G4, G1P4, and G4P4 (for specimen composition, refer to Table 1 and Table 2).

The variations in film morphology resulting from the incorporation of collagen and rapeseed protein hydrolysate into rapeseed meal-based materials were examined by scanning electron microscopy (SEM). The incorporation of hydrolysate induced a transformation in the cross-sectional morphology of the RM-based materials (Figure 7 and Figure S6). Interestingly, the C3 specimen changes from a stratified to a smooth surface morphology, and the A3 material displays a more homogeneous structure.

These morphological changes can be attributed to the high compatibility between the hydrolysate and the protein matrix, which likely explain the reduced water permeability observed in the C3 and A3 specimen [17,52]. In contrast, SEM images of the G1, G4, and G1P4 materials revealed the presence of voids and inter-particle gaps, indicative of a less dense structure and aggregation of protein/peptide molecules, which appear as darker regions on the material surface (Figure S6 Supporting information, page 7) [33,47]. Therefore, the G4P4 material displays a more integrated internal structure, suggesting that it is the optimal formulation for rapeseed meal-based materials.

### 3.8. Aerobic Biodegradation Measurement

**Figure 8:**
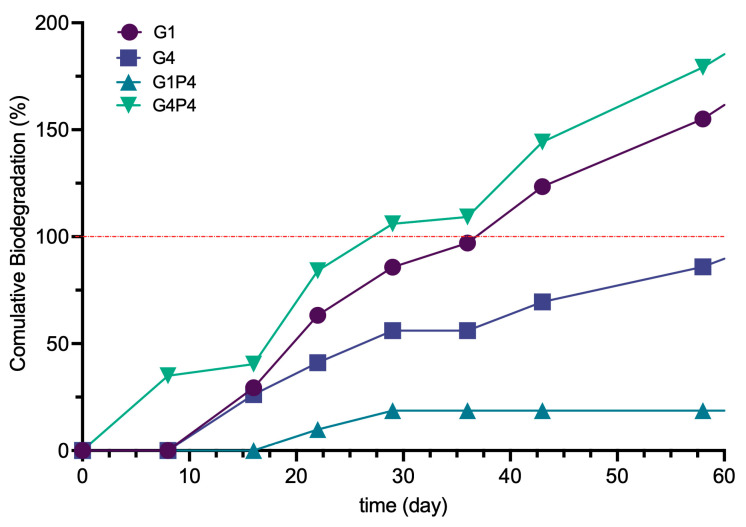
Biodegradability results of specimen G1, G4, G1P4, and G4P4. (For specimen composition, refer to Table 2). The red dotted line refers to 100% biodegradation.

The biodegradation curves (Figure 8) show a three-phase pattern for all the developed materials. A brief lag phase can be observed in the first 10 days, during which the microbial inoculum adapts to the materials. This process is followed by a second degradation phase during which the bacteria utilize the material as a nutrient source for growth. Finally, a plateau phase occurs from days 20 to 40, indicating that the degradation process concluded. Based on these results, it can be stated that biodegradation of the four materials ends under these conditions after 40 days [53]. Among the materials tested, G4P4 demonstrated the highest biodegradability, namely 93% after 43 days, while G1 exhibited a biodegradability of 85%. In contrast, G4 and G1P4 showed substantially lower levels of degradation, with biodegradability of 45% and 19%, respectively, indicating only minimal mass loss for these materials. These results confirm the effect of morphology on moisture absorption and the subsequent properties including biodegradability.

## 4. Conclusions

This study investigated the influence of the incorporation of different weight percentages of hydrolysates of collagen protein and hydrolysates of rapeseed meal proteins on the mechanical properties, fracture morphology, and water absorption of crosslink-stabilized rapeseed meal-based materials.

The incorporation of hydrolysates resulted in the modulation of the mechanical properties of the materials, particularly in terms of flexibility, which increased proportionally with the hydrolysate content. Particularly, the A3 and C3 specimens, containing 0.75% hydrolysate, exhibited lower elastic modulus and higher elongation at break than the control RM material (79.31 N/mm^2^ and 4.35% for A3; 18.24 N/mm^2^, and 6.07% for C3).

The inclusion of hydrolysates allowed us to reduce the glycerol content from 27% to 20%, thanks to replacement with L-proline. Notably, materials containing 0.75% w/wt hydrolysate exhibit reduced water absorption of 85%. The thermal stability of the newly developed materials was improved by covalent crosslinks between the biopolymer chains and epoxide, as revealed by thermogravimetric analysis (TGA).

Apart from some differences among the various C, A, and G formulations, the observed general trend indicates that the modified mechanical response in all cases is due to the reduced density of interchain hydrogen bond crosslinks. This is evidenced by the decrease in elastic modulus and tensile strength while elongation is increased.

The reduction in the crosslink density is reached using low-molecular-weight peptide mixtures. Indeed, collagen and rapeseed hydrolysates, better than glycerol, are capable of interrupting interchain hydrogen bonding among the different macromolecular chains, forming low-viscosity domains in which the protein macromolecules softly crosslinked by the epoxide are partially swollen.

These findings highlight the potential of collagen and rapeseed meal hydrolysate (with high peptide content) to modulate the mechanical properties of protein-based materials, resulting in highly flexible materials that, when molded into slabs, can be easily bent without fatigue and with full recovery of their original shape.

This research establishes new basic knowledge for future exploration of rapeseed meal and other agricultural byproducts as renewable resources for developing a protein-rich material for future potential industrial applications. For instance, the rapeseed meal-based composites developed in this work can be used as controlled-release fertilizer systems, as demonstrated by Versino et al. for cassava-based composites [54]. Such applications represent a sustainable alternative to conventional agrochemicals, contributing to cleaner and more environmentally friendly agriculture. By reducing excessive fertilizer use and mitigating associated environmental issues, particularly nitrate water pollution, these materials offer a promising solution to address critical challenges in modern agriculture.

## Figures and Tables

**Table 1:** Rapeseed meal-based specimen formulation: incorporation of collagen hydrolysate (materials C1–C4) and rapeseed meal protein hydrolysate (materials A1–A4).

	Formulation					
Sample	RM/Casein	Glycerol (%)	Water Solution (%)	Epoxy-Peg	CH (%)	RMH (%)
RM-mat	04:01	27	14	2 × mol Lys.	-	-
C1	04:01	27	14	2 × mol Lys	0.25	-
C2	04:01	27	14	2 × mol Lys	0.50	-
C3	04:01	27	14	2 × mol Lys	0.75	-
C4	04:01	27	14	2 × mol Lys	1.00	-
A1	04:01	27	14	2 × mol Lys	-	0.25
A2	04:01	27	14	2 × mol Lys	-	0.50
A3	04:01	27	14	2 × mol Lys	-	0.75
A4	04:01	27	14	2 × mol Lys	-	1.00

**Table 2:** Rapeseed meal-based specimen formulation: decreased amount of glycerol (G1–G3) and (G4–G5), and addition of proline (G1P4 and G4P4).

	Formulation						
Sample	RM/Casein	Glycerol (%)	Water Solution (%)	Epoxy-Peg	CH (%)	RMH (%)	Pro (%)
RM-mat	04:01	27	14	2 × mol Lys	-	-	-
G1	04:01	20	14	2 × mol Lys	0.75	-	-
G2	04:01	15	14	2 × mol Lys	0.75	-	-
G3	04:01	10	14	2 × mol Lys	0.75	-	-
G4	04:01	20	14	2 × mol Lys	-	0.75	-
G5	04:01	15	14	2 × mol Lys	-	0.75	-
G6	04:01	10	14	2 × mol Lys	-	0.75	-
G1P4	04:01	20	14	2 × mol Lys	0.75	-	7
G4P4	04:01	20	14	2 × mol Lys	-	0.75	7

**Table 3:** Tensile mechanical properties of blends with different concentrations of collagen hydrolysate. Data are presented as an average of three measurements ± of standard deviation.

		Mechanical Properties		
Sample	CH	Stress at Fracture (N/mm^2^)	Elastic Modulus (N/mm^2^)	Elongation at Fracture (%)
RM-mat	/	1.53 ± 0.20	257.71 ± 9.40	1.18 ± 0.28
C1	0.25%	0.57 ± 0.06	38.01 ± 1.04	4.22 ± 0.33
C2	0.50%	0.36 ± 0.11	20.33 ± 1.55	4.78 ± 1.51
C3	0.75%	0.39 ± 0.02	18.24 ± 0.21	6.07 ± 1.03
C4	1.00%	0.35 ± 0.12	19.45 ± 0.89	4.50 ± 0.61

**Table 4:** Tensile mechanical properties of blends with different concentrations of rapeseed meal protein hydrolysate. Data are presented as an average of three measurements ± of standard deviation.

		Mechanical Properties		
Sample	RMH	Stress at Fracture (N/mm^2^)	Elastic Modulus (N/mm^2^)	Elongation at Fracture (%)
RM-mat	/	1.53 ± 0.20	257.71 ± 9.40	1.18 ± 0.28
A1	0.25%	0.79 ± 0.09	57.36 ± 1.15	4.04 ± 0.53
A2	0.50%	0.91 ± 0.16	57.41 ± 2.99	3.97 ± 0.36
A3	0.75%	0.53 ± 0.02	28.86 ± 0.78	4.35 ± 0.33
A4	1.00%	0.93 ± 0.34	79.31 ± 5.79	2.50 ± 0.68

**Table 5:** Tensile mechanical properties of blends with collagen hydrolysate and a decreased concentration of glycerol. Data are presented as an average of three measurements ± of standard deviation.

		Mechanical Properties		
Sample	Glycerol	Stress at Fracture (N/mm^2^)	Elastic Modulus (N/mm^2^)	Elongation at Fracture (%)
C3	27%	0.39 ± 0.02	18.24 ± 0.21	6.07 ±1.03
G1	20%	0.88 ± 0.80	53.17 ± 1.41	4.15 ± 0.16
G2	15%	0.79 ± 0.08	60.13 ± 0.50	3.11 ± 0.49
G3	10%	2.01 ± 0.51	199.40 ± 1.14	2.55 ± 0.43
G1P4	20%	0.52 ± 0.09	23.23 ± 1.12	5.46 ± 0.50

**Table 6:** Tensile mechanical properties of blends with rapeseed meal protein hydrolysate and decreased concentrations of glycerol. Data are presented as an average of three measurements ± of standard deviation.

		Mechanical Properties		
Sample	Glycerol	Stress at Fracture (N/mm^2^)	Elastic Modulus (N/mm^2^)	Elongation at Fracture (%)
A3	27%	0.53 ± 0.02	28.86 ± 0.78	4.35 ± 0.33
G4	20%	0.78 ± 0.12	58.63 ± 1.44	3.42 ± 0.90
G5	15%	1.29 ± 0.84	245.64 ± 4.12	1.89 ± 0.59
G6	10%	1.57 ± 0.89	225.76 ± 26.89	2.32 ± 0.25
G4P4	20%	0.39 ± 0.90	15.08 ±1.12	6.72± 0.46

**Table 7:** Thermal parameters of RM-based materials.

	Thermal Properties		
Sample	Tg (°C)	Tm (°C)	ΔH (J/g)
RM-mat	163.55	186.33	182.48
A3	--------	<160	--------
C3	163.38	185.20	224.17
G1	163.43	186.63	235.86
G4	167.98	176.73	222.74
G1P4	162.97	186.05	228.30
G4P4	--------	<160	--------

## Data Availability

All data supporting the findings of this study are available within the paper and its Supplementary Materials.

## Supplementary Materials

The following supporting information can be downloaded at: https://www.mdpi.com/article/10.3390/polym17131740/s1, Table S1: Proximate analysis of rapeseed meal (provided by Italcol s.r.l.); Table S2: Protein content and Degree of Hydrolysis (DH) of rapeseed meal hydrolysate over the time and scale-up of the enzymatic process; Figure S1: RP-HPLC traces of rapeseed meal protein hydrolysate over time. Alliance Waters 2695 HPLC coupled to a Photodiode Array Detector Waters 2996 (Milford, MA, USA), equipped with a BEH C18 column (1.7 μm, 2.1 × 50 mm) (Des Moines, IA, USA) using solvent systems A (0.1% TFA in H_2_O) and B (0.1% TFA in ACN). Elution was performed with a 15 min linear gradient of 1–80% B (0.1% TFA in ACN), flow rate: 0.5 mL/min, column temperature: 45 °C. The eluates were monitored at 214 nm; Figure S2: TGA curves of RM-based materials with (A) rapeseed meal protein hydrolysate (materials A1-A4); (B) addition of collagen hydrolysates (material C1-C4), (C) decreased amount of glycerol and addition of collagen hydrolysates (material G1) and proline (material G1P4); decreased amount of glycerol and rapeseed meal protein hydrolysate (material G4) and proline (material G4P4); Figure S3: Zoom (150–300 °C and 300–450 °C) of the first derivate curve of TGA curves of RM-based materials with (A) rapeseed meal protein hydrolysate (material A1–A4); (B) addition of collagen hydrolysates (materials C1–C4); (C) decreased amount of glycerol and addition of collagen hydrolysate (material G1) and proline (material G1P4), decreased amount of glycerol and rapeseed meal protein hydrolysate (material G4) and proline (material G4P4); Figure S4: Migration test of (from the top) RM-mat, C3, A3, G1P4, and G4P4 materials at (A) 25 °C for 24 h and (B) 40 °C for 10 days. The solutions were analyzed by UHPLC Thermo Dionex UltiMate 3000 (Thermo Fisher Scientific, Waltham, MA, USA) equipped with an Acquity UPLC BEH C18 column (1.7 μm, 2.1 × 50 mm, Des Moines, IA, USA) using solvent systems A (0.1% formic acid in H_2_O) and (B) (0.1% formic acid in ACN). Elution was performed with an 8-min linear gradient from 1% to 95% phase B at a flow rate of 0.5 mL/min at 35 °C. The eluates were monitored at 214 nm to detect the presence of protein or peptides in the eluate; Figure S5: (A) DSC curve of RM-mat, C3, G1, G4, G1P4 materials; (B) Zoom (150–175 °C) of DSC curve of RM-mat, C3, G1, G4, G1P4 materials; Figure S6: Scanning Electron Microscopy images of cryofracture cross-section of specimens (from the top) RM-mat, C3, A3, G1, G4, G1P4, and G4P4; Figure S7: (A) Schematic view of biodegradation setup according to AST D5988; (B) Biodegradation setup applied in current research.

## Abbreviations

The following abbreviations are used in this manuscript:

ACN	acetonitrile;
CH	collagen hydrolysate;
ΔH	melting enthalpy;
DSC	differential scanning calorimetry;
Epoxy-PEG	poly-(ethylene glycol)diglycidyl ether;
LDPA	low-density polyethylene;
PBSA	Poly-(butylene succinate-co-adipate);
PCL	polycaprolactone;
PVA	polyvinyl alcohol;
PVC	polyvinlylchloride;
RM	rapeseed meal;
RMH	rapeseed meal hydrolysate;
RM-Mat	reference material
RP-HPLC	reverse-phase high-performance liquid chromatography;
SDS	sodium dodecyl sulfate;
SDS-PAGE	sodium dodecyl sulfate-polyacrylamide gel electrophoresis;
SEM	scanning electron microscopy;
TFA	trifluoroacetic acid;
TGA	thermogravimetric analysis;
Tg	glass transition temperature;
Tm	melting temperature;
UHPLC	ultra-high-performance liquid chromatography;
WPI	whey protein isolates.
