# Supplementary material for: Incorporation of Protein Hydrolysate into Rapeseed Meal-Based Materials to Improve Flexibility
Source: Polymers (Basel). 2025 Jun 22;17(13):1740. doi: 10.3390/polym17131740 (PMC12252457; doi:10.3390/polym17131740)
Supplement: Supplementary file 1 [file polymers-17-01740-s001.zip › polymers-3711731-supplementary.pdf]

SUPPORTING INFORMATION

Incorporation of Protein Hydrolysate into Rapeseed Meal-Based Materials to Improve Flexibility

Sara Aquilia [1,2,3], Claudia Bello [1,2], Michele Pinna [3], Sabrina Bianchi [3], Walter Giurlani [2], Francesco Ciardelli [3], Luca Rosi [2*] and Anna Maria Papini [1,2*]

CONTENT:

Table S1: Proximate analysis of rapeseed meal (provided by Italcol s.r.l.)
Table S2: Protein content and Degree of Hydrolysis (DH) of rapeseed meal hydrolysate over the time and scale-up of the enzymatic process.
Figure S1: RP-HPLC traces of rapeseed meal protein hydrolysate over time. Alliance Waters 2695 HPLC coupled to a Photodiode Array Detector Waters 2996 (Milford, MA, USA), equipped with a BEH C18 column (1.7 µm, 2.1 × 50 mm) (Des Moines, IA, USA) using solvent systems A (0.1% TFA in H_2O) and B (0.1% TFA in ACN). Elution was performed with a 15 min linear gradient of 1% - 80% B (0.1% TFA in ACN), flow rate: 0.5 mL/min, column temperature: 45 °C. The eluates were monitored at 214 nm.
Figure S2: TGA curves of RM-based materials with A) rapeseed meal protein hydrolysate (materials A1-A4); B) addition of collagen hydrolysates (materials C1-C4); C) decreased amount of glycerol and addition of collagen hydrolysates (material G1) and proline (material G1P4), decreased amount of glycerol and rapeseed meal protein hydrolysate (material G4) and proline (material G4P4).
Figure S3: Zoom (150-300 °C and 300-450 °C) of the first derivate curve of TGA curves of RM-based materials with A) rapeseed meal protein hydrolysate (materials A1-A4); B) addition of collagen hydrolysates (materials C1-C4); C) decreased amount of glycerol and addition of collagen hydrolysate (material G1) and proline (material G1P4), decreased amount of glycerol and rapeseed meal protein hydrolysate (material G4) and proline (material G4P4).
Figure S4: Migration test of (from the top) RM-mat, C3, A3, G1P4, and G4P4 materials at A) 25 °C for 24 h and B) 40°C for 10 days. The solutions were analyzed by UHPLC Thermo Dionex UltiMate 3000 (Thermo Fisher Scientific, Waltham, MA, USA) equipped with an Acquity UPLC BEH C18 column (1.7 µm, 2.1 × 50 mm, Des Moines, IA, USA) using solvent systems A (0.1% formic acid in H_2O) and B) (0.1% formic acid in ACN). Elution was performed with an 8-min linear gradient from 1% to 95%, phase B at a flow rate of 0.5 mL/min at 35 °C. The eluates were monitored at 214 nm to detect the presence of protein or peptides in the eluate.
Figure S5: A) DSC curve of RM-mat, C3, G1, G4, G1P4 materials; B) Zoom (150-175 °C) of DSC curve of RM-mat, C3, G1, G4, G1P4 materials.
Figure S6: Scanning Electron Microscopy images of cryofracture cross-section of specimens (from the top) RM-mat, C3, A3, G1, G4, G1P4, and G4P4.
Figure S7. A) Schematic view of biodegradation setup according to AST D5988; B) Biodegradation setup applied in current research.

Table S1. Proximate analysis of rapeseed meal (provided by Italcol s.r.l.)

	**Rapeseed meal (g/100g)**	**LD**
Crude Ash	6.58	0.05
Crude Oil and fats (Crude Lipids)	2.3	0.1
Moisture	13.5	0.1
Crude Fiber (cellulose)	13.6	0.1
Crude Protein (N × 6.25)	32.3	0.1
Nitrogen-insoluble extractives (p.d. to 100)	31.7	-
Ash insoluble in HCl	0.34	0.05

Table S2. Protein content and Degree of Hydrolysis (DH) of rapeseed meal hydrolysate over the time and scale-up of the enzymatic process

Rapeseed meal hydrolysate	Protein content (mg/mL)	DH (%)	Y (%)
1h	0.41	1.42	13.56
2h	0.59	3.86	19.67
4h	1.19	11.85	39.64
6h	1.31	13.49	43.74
8h	0.67	4.96	22.42
Overnight	1.09	10.57	36.45
Scale up	1.85	20.65	46.22

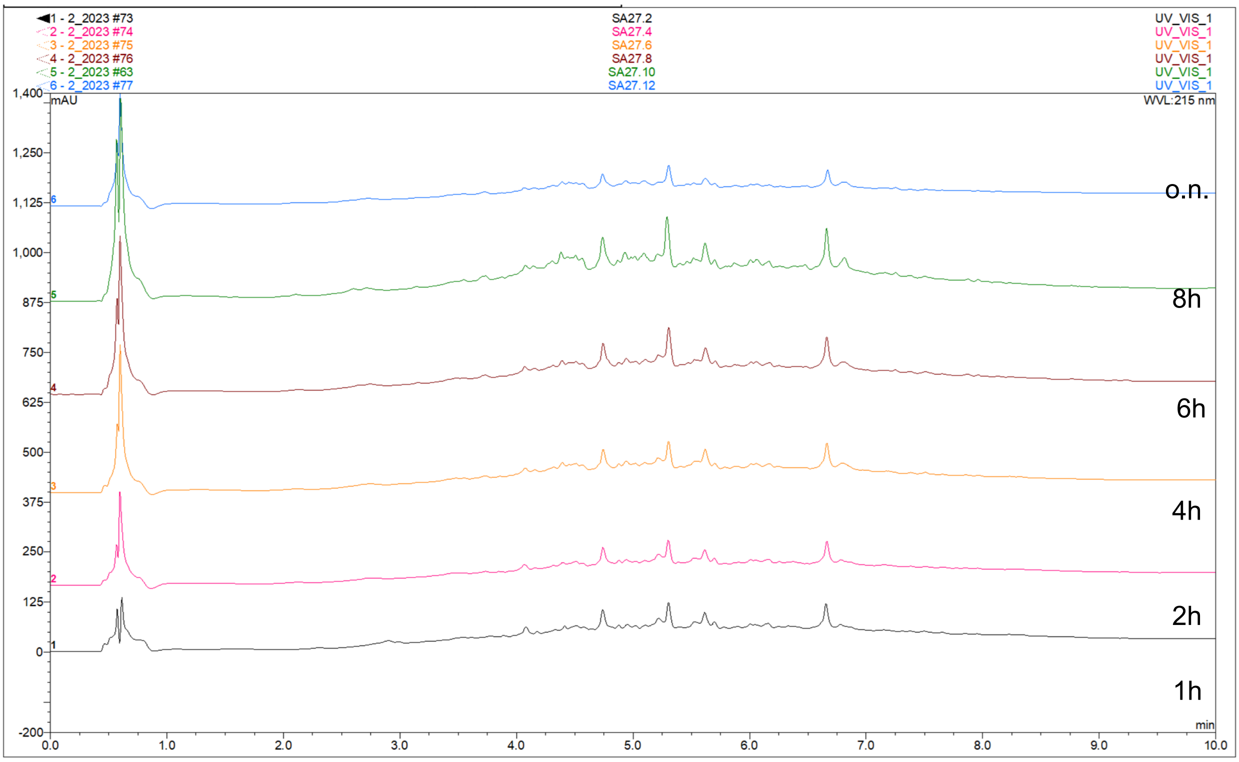

Figure S1. RP-HPLC traces of rapeseed meal protein hydrolysate over time. Alliance Waters 2695 HPLC coupled to a Photodiode Array Detector Waters 2996 (Milford. MA, USA), equipped with a BEH C18 column (1.7 µm, 2.1 × 50 mm) (Des Moines, IA, USA) using solvent systems A (0.1% TFA in H_2O) and B (0.1% TFA in ACN). Elution was performed with a 15 min linear gradient of 1% - 80% B (0.1% TFA in ACN), flow rate: 0.5 mL/min, column temperature: 45 °C. The eluates were monitored at 214 nm.

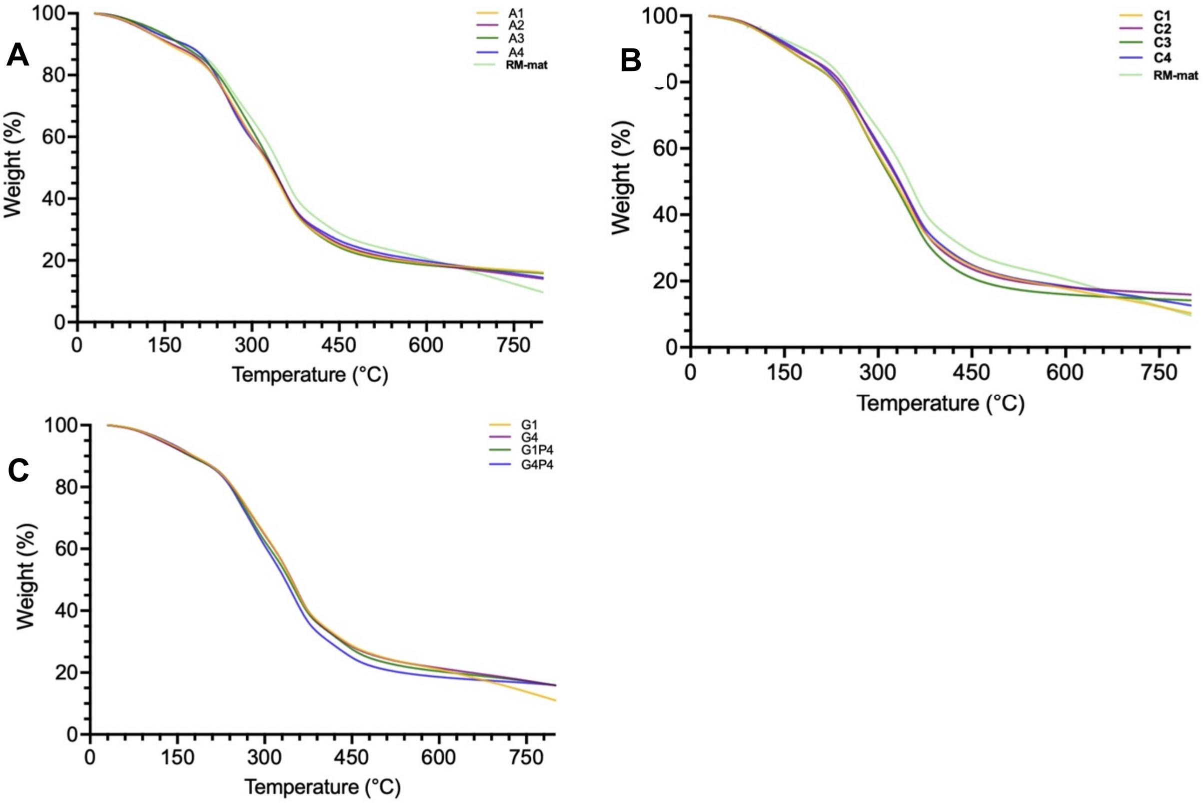

Figure S2. TGA curves of RM-based materials with A) rapeseed meal protein hydrolysate (materials A1-A4); B) the addition of collagen hydrolysates (materials C1-C4); C) decreased amount of glycerol and addition of collagen hydrolysates (material Gl) and proline (material G1P4), decreased amount of glycerol and rapeseed meal protein hydrolysate (material G4) and proline (material G4P4).

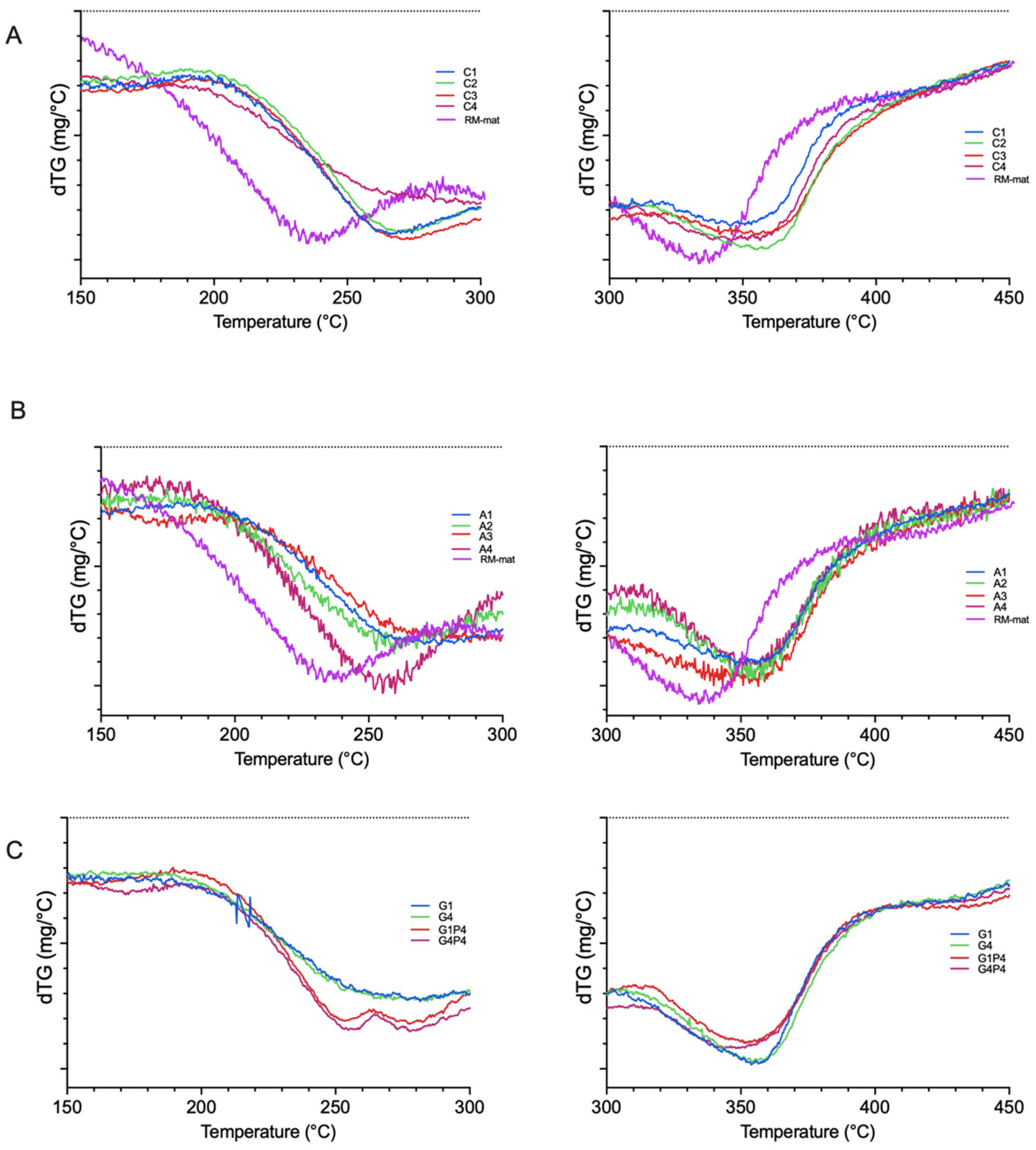

Figure S3. Zoom (150-300 °C and 300-450 °C) of the first derivate curve of TGA curves of RM-based materials with A) rapeseed meal protein hydrolysate (materials A1-A4); B) addition of collagen hydrolysates (materials C1-C4); C) decreased amount of glycerol and addition of collagen hydrolysate (material G1) and proline (material G1P4), decreased amount of glycerol and rapeseed meal protein hydrolysate (material G4) and proline (material G4P4).

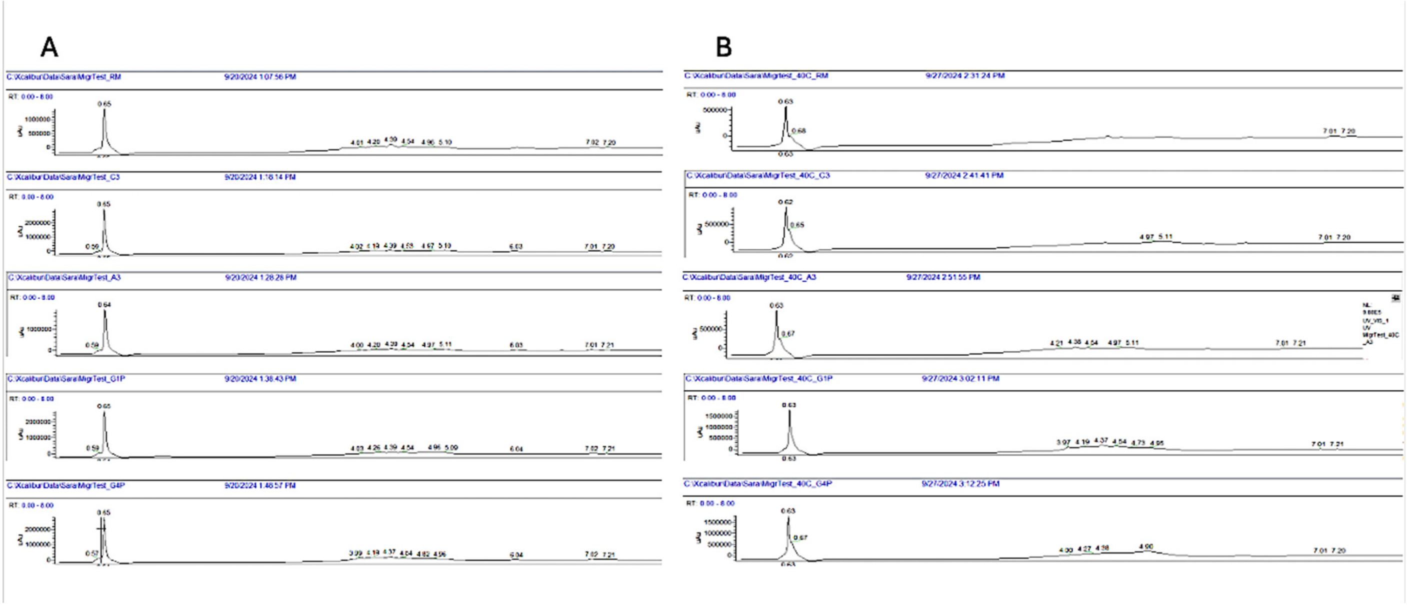

Figure S4. Migration test of (from the top) RM-mat, C3, A3, G1P4, and G4P4 materials at A) 25 °C for 24 h and B) 40°C for 10 days. The solutions were analyzed by UHPLC Thermo Dionex UltiMate 3000 (Thermo Fisher Scientific, Waltham, MA, USA) equipped with an Acquity UPLC BEH C18 column (1.7 µm, 2.1 × 50 mm, Des Moines, IA, USA) using solvent systems A (0.1% formic acid in H_2O) and B) (0.1% formic acid in ACN). Elution was performed with an 8-min linear gradient from 1% to 95%, phase B at a flow rate of 0.5 mL/min at 35 °C. The eluates were monitored at 214 nm to detect the presence of protein or peptides in the eluate.

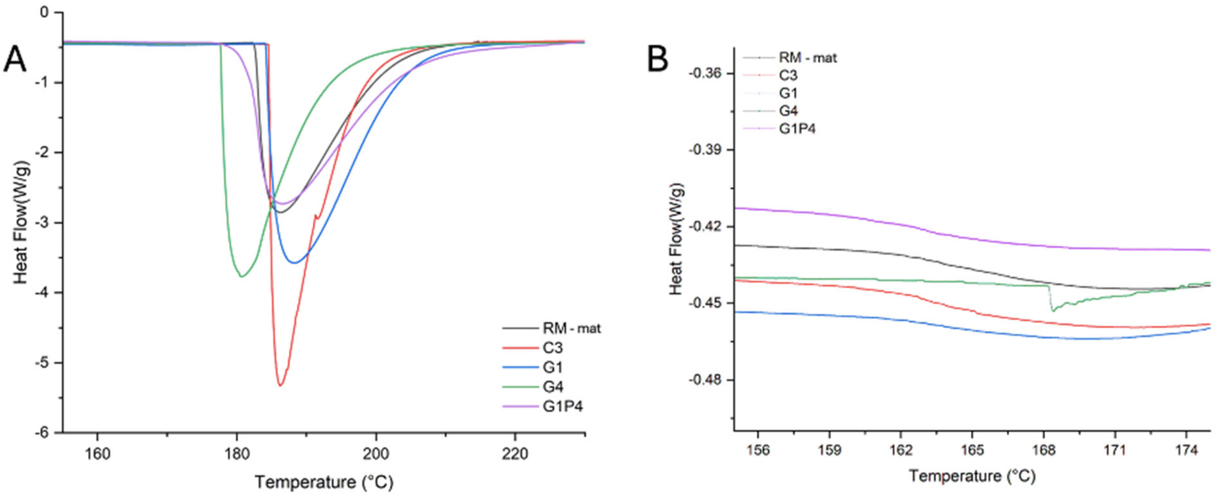

Figure S5. A) DSC curve of RM-mat, C3, G1, G4, G1P4 materials; B) Zoom (150-175 °C) of DSC curve of RM-mat, C3, G1, G4, G1P4 materials.

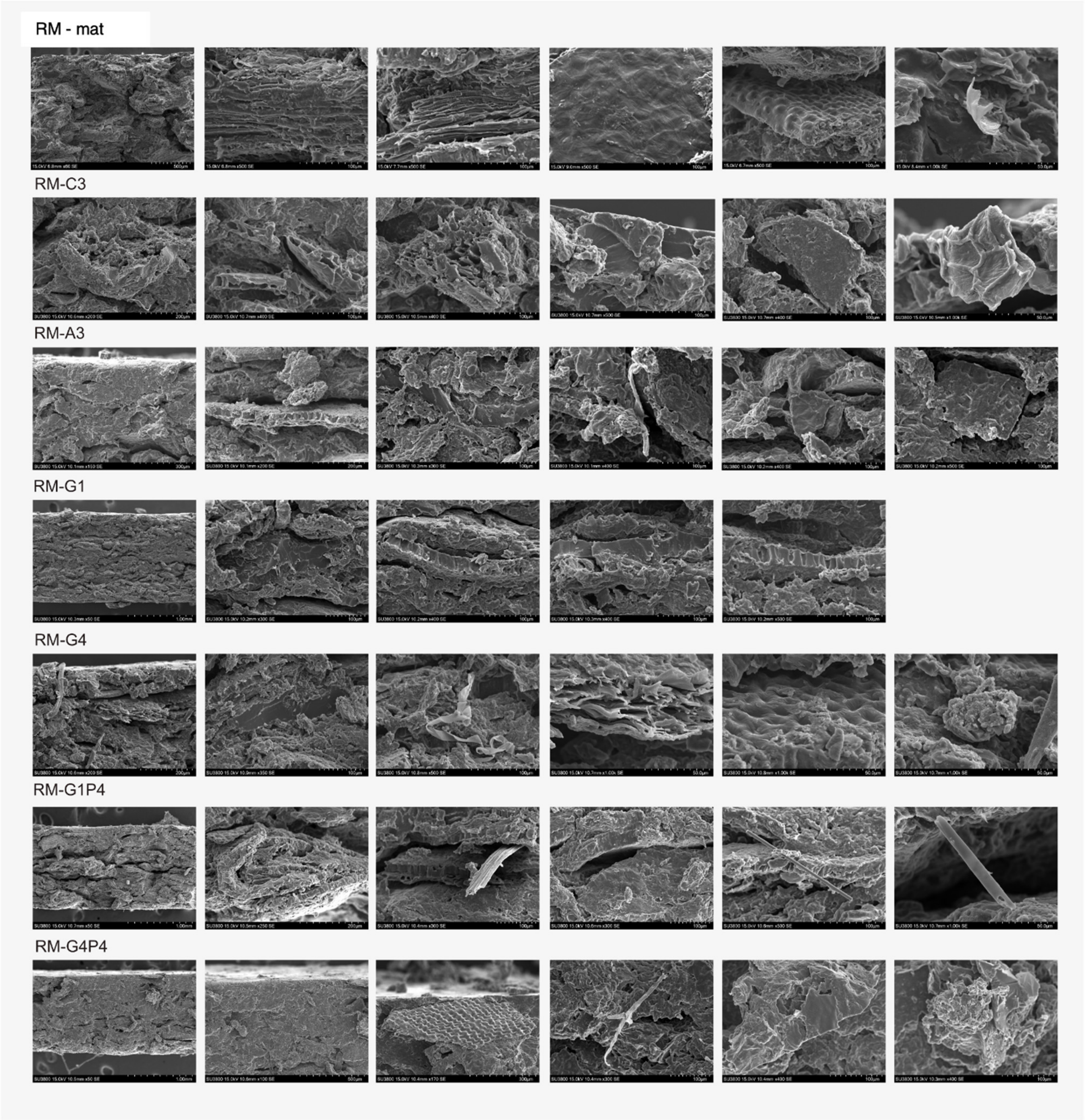

Figure S6. Scanning Electron Microscopy images of cryofracture cross-section of specimens (from the top) RM-mat, C3, A3, G1, G4, G1P4, and G4P4.

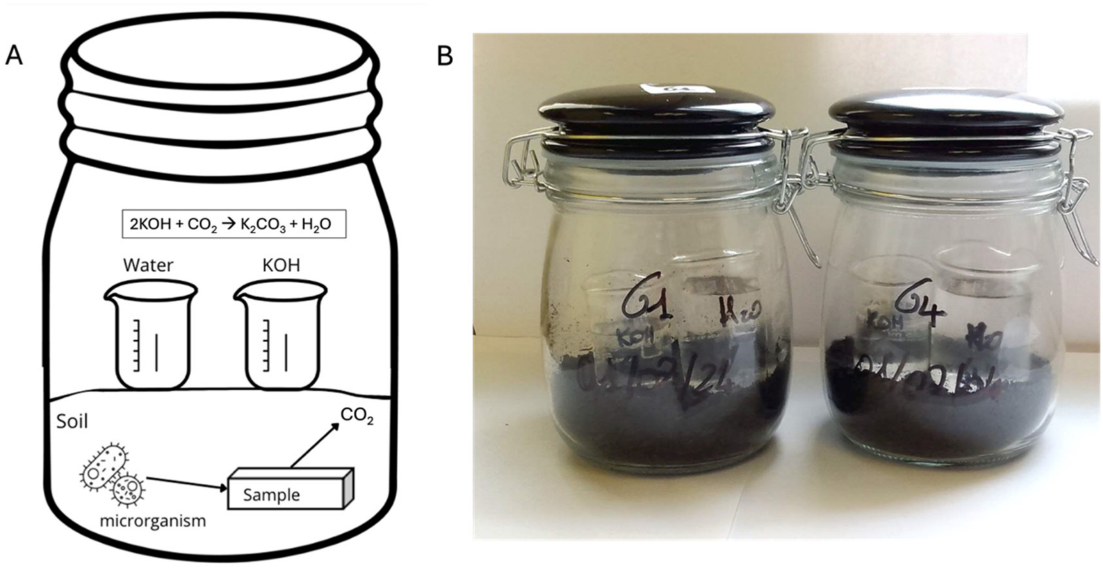

Figure S7. A) Schematic view of biodegradation setup according to AST D5988; B) Biodegradation setup applied in current research.
